# The effects of sleep deprivation and extreme exertion on cognitive performance at the world-record breaking Suffolk Back Yard Ultra-marathon

**DOI:** 10.1371/journal.pone.0299475

**Published:** 2024-03-14

**Authors:** Sandy Benchetrit, Juan I. Badariotti, Jo Corbett, Joseph T. Costello

**Affiliations:** 1 Extreme Environments Thematic Research Group, School of Sport, Health & Exercise Science, University of Portsmouth, Portsmouth, United Kingdom; 2 Gloucestershire Hospitals NHS Foundation Trust, Gloucestershire Royal Hospital, Gloucester, United Kingdom; University of Mississippi, UNITED STATES

## Abstract

Using a prospective observational design, this study investigated the hypothesis that competing in the Suffolk Back Yard Ultra-marathon, would result in impaired cognitive performance and examined whether pre-race sleep patterns could mitigate this. Fifteen runners (1 female) volunteered to undertake this study and eleven males were included in the final analysis. Before the race and after withdrawal participants completed the following cognitive performance tasks: 2 Choice Reaction Time (2CRT), Stroop, and the Tower Puzzle. Pre-race sleep strategies were subjectively recorded with a 7-day sleep diary. Following race withdrawal, reaction time increased (Δ 77±68 ms; p = 0.004) in the 2CRT and executive function was impaired in the Stroop task (Interference score Δ -4.3±5.6 a.u.; p = 0.028). Decision making was not affected in the Tower Puzzle task. There was a significant correlation between the pre-race 7-day average sleep scores and both 2CRT Δ throughput (r = 0.61; p = 0.045) and 2CRT Δ RT (r = -0.64; p = 0.034). This study supports the hypothesis that running an ultra-marathon, which includes at least one night of sleep deprivation, impairs cognitive performance and provides novel evidence suggesting good sleep quality, in the week prior to an ultra-marathon, could minimise these effects.

## 1. Introduction

Ultra-marathons, running races which exceed the 42.2 km distance of a marathon, have increased in popularity over the last decade [1, 2]. With the motivation for these often being the exploration of the mental and physical limits of the self, it is no surprise that these events are becoming more extreme and increasing in length to exceptionally long distances [2–4]. Consequently, fatigue and sleep deprivation often form a significant part of the challenge of these events. Unfortunately, there is limited research examining ways to mitigate the effects of restricted sleep during an ultra-marathon.

Overall race performance is dependent on both race specific variables and individual factors, therefore an assessment of the impact of pre-race sleep strategies based solely on this outcome could under value the importance of sleep management strategies at the individual level. These endurance events are often located in extreme and remote environments requiring complex skills (e.g. navigational skills). Runners are therefore required to maintain attention, concentration and vigilance in order to react to changes in their environment and make safe decisions. Studies on complete sleep deprivation have typically demonstrated a negative impact on cognitive performance, specifically reaction time, and mood, in healthy individuals [5–7]. However, the combined effect of arduous exercise and sleep deprivation on cognitive performance is comparatively less well examined and could highlight ways to improve individual performance and safety. Research on endurance sports in solo sailors [8], cyclists [9] and military events [10] has demonstrated lapses in attention, slower response times, poor mood and technical errors following sleep deprivation. Although the current literature base focusing on ultra-marathons has also shown conflicting results [11–13], races involving sleep deprivation have consistently reported a deterioration in cognitive performance [14–17]. However, few studies have assessed the impact of pre-race sleeping strategies on cognitive performance during ultra-marathons. Hurdiel and colleagues [16] demonstrated impaired cognitive performance was associated with in-race sleeping during a 168 km race. They postulated that napping was an indication of extreme fatigue and not sufficient in duration to mitigate the detrimental effects on cognitive performance [16]. Poussel and colleagues [18] found sleep extension prior to the race led to faster finish times compared to the use of sleep restriction training and daytime naps, but did not examine cognitive performance *per se*. Interestingly, in a questionnaire based study Martin and colleagues [19] found 73.9% of ultra-marathon runners altered their sleep prior to a race with 54.7% attempting to extend their sleep. To the best of our knowledge, no studies have investigated the impact of sleeping habits prior to an ultra-marathon on cognitive performance. Improved knowledge of the complex relationship between sleep deprivation and endurance exercise on the cognitive performance of ultra-runners could further elucidate the effects of such significant stressors and lead to the development of sleep strategies to improve race performance and safety. Accordingly, the present study sought to examine the effects of running an ultra-marathon, incorporating at least one night of sleep deprivation, on the cognitive performance of runners and its association with pre-race sleeping strategies. It was hypothesised that (1) cognitive performance would deteriorate after the ultra-marathon; and that (2) pre-race sleeping patterns characterised by high sleep quality and quantity would be protective against these effects.

## 2. Methods

The present prospective observational study examined cognitive performance in runners before and after completing the Suffolk Back Yard Ultra-marathon (SBYU) on the 5^th^– 7^th^ June 2021. This study adhered to the standards set by the *Declaration of Helsinki*, except for registration in a database, and was approved by the Science & Health Faculty Ethics Committee of the University of Portsmouth (reference number: 2021–037). All participants gave their written informed consent before data collection. Three authors had access to participant names to collect data during the race. All collected data were anonymised using a linked-anonymised key spreadsheet applying a research number to each participant for data analysis.

### 2.1 Participants

**Fig 1:**
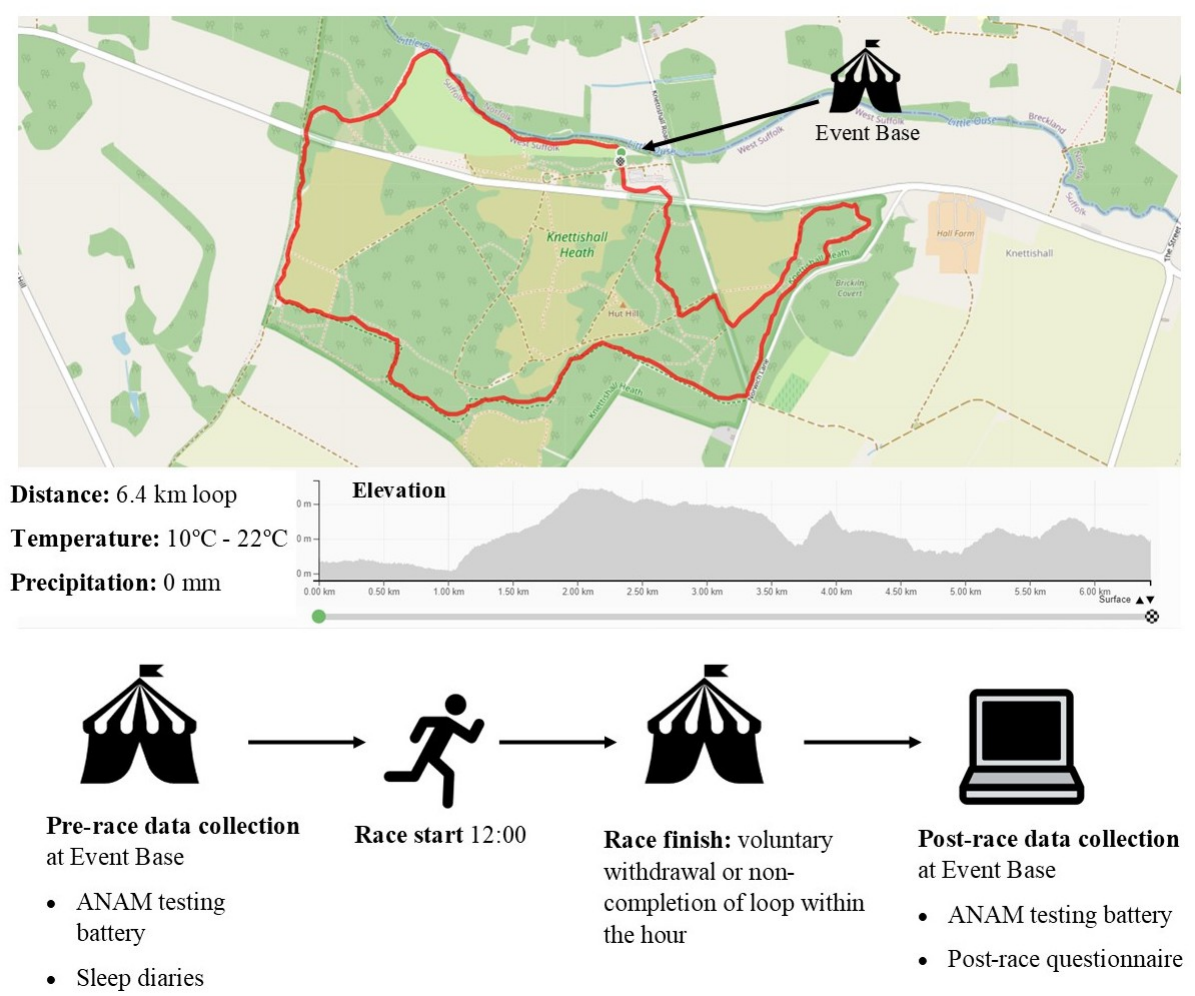
Suffolk Back Yard Ultra running route and data collection time points. Weather data for 5th June to 7th June 2021. Image reproduced with permission from gpx.studio. Base map data source from © OpenStreetMap contributors, available under the Open Database Licence (openstreetmap.org). Gpx data file used with permission from © Challenge Running.

The SBYU is a non-stop Backyard ultra-running race, based at Knettishall Heath Country Park in Suffolk (Fig 1). It commenced at 1200 (midday) on the 5^th^ June and participants ran a single 6.4 km loop on the hour, every hour until voluntary withdrawal or until they were unable to complete a lap within the hour. The race ended once only one runner remained to complete a solo loop. The Backyard Ultra World Record, at the time, was broken at the event, with one individual (not one of the participants in the current study) completing 81 hours and 542 km. Prospective participants were invited to take part in the study if they were already registered to run the SBYU, aged 18 years or over, and aimed to run into the night (past 0300). The inclusion criterion of running until 0300 was chosen to capture runners who experienced a minimum of 16 hours of sleep deprivation (latest estimated wake-up at 1100). While the effects of sleep deprivation on cognitive performance increase with exposure time, studies have demonstrated a measurable impact after a minimum of 16 hours [20]. Recruitment commenced on the 27^th^ April 2021 and ended on the 25^th^ May 2021. A convenience sample of 16 volunteers (15 males, 1 female) provided their consent to partake in the study. Demographical and medical history was assessed with a questionnaire. All participants were non-smokers and free of significant cardiovascular, respiratory or neurological disease. Two participants had a history of mild asthma, for which neither required regular medications nor inhalers. Two volunteers had chronic hip and lower limb injuries. Participants diagnosed with a sleeping disorder or taking regular prescribed medication for insomnia were excluded. All participants were UK based.

On the morning of the race, one individual decided not to undertake the race. Therefore, 15 participants (1 female) [mean ± SD, age: 40 ± 8.6 years, mass: 75.1 ± 8.5 kg, height: 177 ± 8.3 cm, body mass index: 23.8 ±2.1 kg.m^-2^, average weekly distance run: 76.9 ± 37.7 km, number of ultra-marathons completed: 8.6 ± 10.6] completed familiarisation and baseline cognitive battery testing prior to the race start. This was completed in a seated position in front of laptop computers under shade with instructions to avoid all distractions for the duration of the testing. Immediately following withdrawal from the race, participants completed the post-race Automated Neurophysiological Assessment Metrics (ANAM) cognitive testing battery and post-race questionnaire (described later). The research station was positioned at the finish line and only participants who completed the testing on race completion prior to sleeping were included in the final analysis (median [IQR] time of commencing the cognitive battery testing after race completion was 9 [12] min).

### 2.2 Outcome measures

#### 2.2.1 Cognitive performance measures

Cognitive performance was assessed using the ANAM (version 4) General Neuropsychological Screening (GNS) battery, which has previously been used to measure cognition in a variety of contexts, including in extreme environments, such as hypoxia [21] and in military training [22] and has good construct validity [23] and minimal learning effect after the second administration [24, 25].

The specific battery used was designed to reflect existing literature in this field and the importance of various cognitive domains in running safety and performance. Studies assessing cognitive performance in ultra-marathon field studies have used a plethora of diverse tools to assess cognitive performance. These include language control [26], problem solving [13], concentration and attention [13, 16, 17], reaction times [11, 15] and executive function [11, 12, 15, 26], with the latter three most frequently studied. The Stroop task has frequently been used as a measure of executive function in studies of cognitive performance in endurance events (two ultra-marathons [11, 12] and one adventure race [27]) and in laboratory studies looking into the effects of sleep deprivation on athletes [28]. Reaction time has been used as an outcome measure both in field studies and in laboratory studies examining the impact of sleep deprivation in athletes [5, 15, 29]. Studies on the effect of sleep deprivation on cognitive performance have suggested that the tasks most affected are those performed by the prefrontal cortex, such as temporal memory and response inhibition [30]. Therefore, the individual tasks selected were 2 Choice Reaction Time (2CRT), Stroop, and the Tower Puzzle. A practice effect has previously been reported using ANAM, however this effect was found to be most significant at the second application of the tasks with minimal change after three trials [25]. Therefore, following manufacturers guidelines, a thorough familiarisation of the battery (2CRT, Stroop and Tower Puzzle) was completed three times on the morning of the race and the fourth attempt was used as the baseline, pre-race, measure. The three tasks were undertaken in the same order both during familiarisation and testing. Each set of three tasks were designed to take no longer than 10 min combined, therefore a maximum of 40 min for familiarisation and baseline measurements and 10 min for post-race testing.

The 2CRT task assesses processing speed, attention and concentration, along with a motor speed component [31]. Participants were required to press a specific key as soon as possible when they saw a “*” symbol and a different key when an “O” symbol appears in 60 trials per task. The outcome measures recorded were Reaction Time (ms), Accuracy (% correct) and Throughput (the number of correct responses per minute of available response time), a measure of cognitive efficacy which combines both reaction time and accuracy into a single metric [31].

The Stroop task is a measure of executive function and assesses selective attention, inhibitory control and processing speed [31]. It has three levels of 60 trials, in the first, the words “RED”, “GREEN” and “BLUE” are presented separately in white on a black background. Participants were instructed to select a specific key for each word. In the second level a series of colored “XXXX” appeared on the screen either in red, green or blue. Participants were instructed to select the key corresponding to that color. In the third level the individual words were presented in a color which didn’t match the name of the color depicted, for example the word “RED” was displayed colored in blue. Participants were asked to press the key assigned to the color and not the word. The primary outcome measures were Level 3 accuracy (%), Level 3 Reaction Time (ms) and the Interference Score (number of correct responses on level 3 minus the Predicted Color/Word Score), which reflects inhibitory control, the ability to ignore an automatic response to carry out a different action, and executive function [11].

The Tower Puzzle assesses decision making, planning, working memory and visuo-spatial ability [31]. There are five tests of increasing difficulty. Each test is composed of three posts, each with a stack of different sized disks, on the screen. Participants must move all the disks to the center post in size order, with the largest at the bottom and the smallest at the top. However, only one disk can be moved at a time and can only be placed on a larger disk. This must be done in as few moves as possible. The primary outcome measures were the Mean Reaction Time (ms), Mean Score (a composite score of Accuracy, Speed, Problem Difficulty and Range Constant) and Move Ratio (Actual Moves for all puzzles minus Errors for all puzzles, divided by the Minimum Moves for all puzzles). For further explanation, examples and images of the ANAM tasks discussed above are described by Reeves and colleagues [32].

#### 2.2.2 Secondary outcome measures

Secondary outcome measures included subjective measures of sleep and adverse events. In the 7 days leading up to the race, participants completed the Loughborough daily sleep diary, which has frequently been used in research and is recommended in clinical practice [33]. Outcome measures used included total time in bed (h), time asleep (h) and quality of sleep (based on a 5-point Likert scale) in the night before and as a 7-day average before the race. The post-race questionnaire collected data on race performance, adverse events during the race and in-race sleeping.

### 2.3 Statistical analyses

The distribution of data was assessed using descriptive methods (skewness, outliers, and distribution plots) and inferential statistics (Shapiro—Wilk test). Where normal distribution was violated non-parametric analyses were performed. Pre- and post-race cognitive performance and sleep outcomes were assessed using a two-sided paired sample T-test or the Wilcoxon signed rank test where appropriate. Effect sizes were calculated using Cohen’s d (using the standard deviation of the difference) and considered small d = 0.2, medium d = 0.5 and large d = 0.8 [34]. Pearson’s (parametric data) or Spearman’s correlations (non-parametric data) were used to determine the correlation between sleep outcomes (total sleep time and quality of sleep) and the change in cognitive task performance (ΔRT, Δaccuracy, Δthroughput). Both Pearson’s and Spearman’s correlation coefficients were considered as strong (≥0.70), moderate (0.40–0.69), and weak (0.1–0.39) [34]. All statistical analyses were performed using IBM SPSS Statistics version 26.0 (IBM Corp., Armonk, NY, USA). A post hoc power analysis (1-β) was conducted using G*Power version 3.1.9.6 (Franz Faul, Universität Kiel, Germany). Data are presented as mean (SD) or as median and 25th and 75th percentiles (IQR) unless otherwise stated. Statistical significance was accepted at p<0.05.

## 3. Results

### 3.1 Participants

One participant did not complete post-race testing and 3 participants withdrew before 0300 am on the 6^th^ June. Therefore, 11 participants were included in the final analysis (time running 23.8 ± 7.9 hours, distance 158.5 ± 53.8 km). Ambient temperature ranged from 10–22°C over the course of the event and there was no rainfall recorded.

### 3.2 Cognitive performance

Reaction time, accuracy, and throughput data are displayed in Table 1. Reaction time increased (worsened) post-race across all three tasks (all p<0.05). Throughput in the 2CRT task, level 3 accuracy and the interference score in the Stroop task all decreased (worsened) post-race (Table 1). No other significant changes were observed in cognitive performance.

**Table 1:** Cognitive performance before and after the Suffolk backyard ultra-marathon (N = 11).

	Pre-race	Post-race	Δ	p-value	Cohen’s d^b^	1-β
**2 Choice Reaction Time**						
Accuracy (%)^a^	97.5 [2.5]	100 [2.5]	0 [2.5]	0.577	0.19 [-0.42–0.78]	0.08
Reaction Time (ms)	452 [38]	529 [81]	77 [68]	**0.004**	1.09 [0.33–1.81]	0.95
Throughput (a.u.)	131.0 [12.1]	113.9 [16.6]	-17.0 [11.4]	**<0.001**	1.17 [0038–1.93]	0.94
**Stroop Task**						
Level 3 accuracy (%)	94.4 [5.3]	91.9 [4.4]	-2.5 [2.8]	**0.014**	0.52 [-0.12–1.15]	0.34
Level 3 Reaction Time (ms)	809 [213]	900 [208]	91 [119]	**0.029**	0.77 [0.79–1.44]	0.25
Interference Score (a.u.)	23.1 [9.3]	18.8 [6.8]	-4.3 [5.6]	**0.028**	0.53 [-0.12–1.15]	0.36
**Tower Puzzle**						
Mean Reaction Time (ms)^a^	1680 [478]	2394 [736]	517 [296]	**0.003**	1.75 [0.77–2.69]	0.99
Mean Score (a.u.)^a^	2332.6 [624.4]	2174.4 [620.8]	-105.1 [303.5]	0.248	0.25 [-0.35–0.85]	0.11
Move Ratio (a.u.)^a^	1.08 [0.12]	1.21 [0.36]	0.07 [0.17]	0.240	0.42 [-0.20–1.03]	0.15

Cognitive performance measures are reported as mean [SD].

^a^data are median [IQR]. All Δ data are mean [SD].

^b^data are effect size [95% CI].

### 3.3 Sleep measures

Total average time asleep in the 7 days before the race was higher compared to the night prior to the race (7.29 ± 0.96 hours, 6.78 ± 1.32 hours respectively, p = 0.042, d = 0.44). There was no statistical difference in other measured outcomes; participants spent 8.26 ± 0.99 hours in bed the night prior to the race and rated their sleep quality 3.45 ± 0.93 out of 5. In comparison, in the 7 nights prior to the race participants spent 8.31 ± 1.30 hours in bed (p = 0.803) and rated their sleep quality 3.68 ± 0.58 out of 5 (p = 0.355).

Questionnaire results showed eight participants intentionally altered their normal sleeping patterns in the week prior to the race (Table 2). Six increased their sleep by 30 min to 2 hours each night for at least two nights prior to the race and five napped during the day in the week prior to the race. One participant decreased their sleep by 1h due to work related travel requirements but attempted to counter this by napping 20–60 minutes during the day. Interestingly the two participants who ran the shortest distance did not alter their sleep patterns prior to the race (Table 2). On the other hand, the three participants who did not alter their sleep were among the top six with the smallest pre-post change in cognitive performance.

**Table 2:** Sleep alterations in the week prior to the race listed in descending order of distance covered during the race.

Participant	Distance run (km)	Night time sleep alterations	Day time naps
1	248	none	0–30 min nap once per day
2	228	Increased sleep by 1h per night	None
3	208	Increased sleep by 30min for 2 nights	0–30 min nap once per day
4	201	Decreased sleep by 1h per night	20–60 min nap once per day
5	168	None	None
6	127	Increased sleep by 1h per night	20–60 min nap 3x per day
7	121	Increased sleep by 30-60min for 3 nights	0–30 min nap once per day
8	121	Increased sleep by 2h for 2 nights	None
9	114	Increased sleep by 1h for 4 nights	None
10	107	None	None
11	101	None	None

Two participants ‘napped’ during the event (range: 1–15 minutes) and were among the four research participants who exceeded the 200 km distance.

### 3.4 Adverse events

Eight participants reported adverse events during the ultra-marathon (13 separate events). Four participants fell (36%), two experienced hallucinations (18%), three became injured (27%) and four developed difficulties coordinating their movements (ataxia) (36%). While the sample size is too small to correlate adverse events with race and cognitive performance, of the top five performing participants (greatest distance covered) only two adverse events (two falls) were reported. Interestingly, the participant with the largest decrease in cognitive function in all tasks reported three separate adverse events (injury, ataxia and hallucinations). The five participants with the greatest decline in the Interference Score and 2CRT Throughput reported a greater number of adverse events compared to the top six participants (8 adverse events, 62%).

### 3.5 Correlations

**Fig 2:**
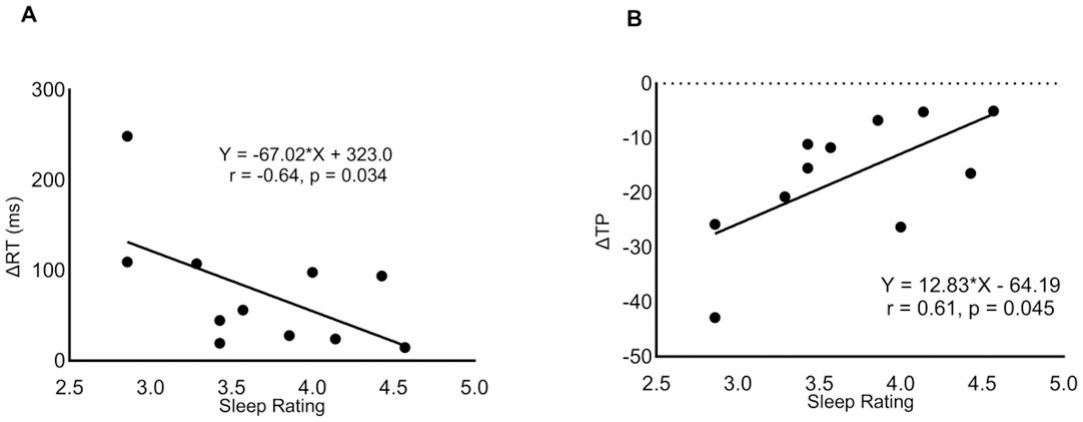
Simple linear regression models of the average Sleep Rating in the 7 days prior to the race versus the pre- to post-race changes in cognitive performance in the 2 Choice Reaction Time (ΔRT) (A) and Throughput (ΔTP) (B) (n = 11). Linear regression equations and Spearman’s correlation coefficients displayed in each figure.

A moderate negative association was observed between the average sleep ratings over 7 days prior to the race and the 2CRT ΔRT (r = -0.64, p = 0.034) (Fig 2a). There was a moderate association between the average sleep ratings over 7 days and the 2CRT ΔTP (r = 0.61, p = 0.045) (Fig 2b). There was also a correlation between the total average time asleep and average sleep rating in the 7 days prior to the race (r = 0.70, p = 0.016). There was no significant correlation between the total distances covered and cognitive performance or sleep outcomes. No other significant correlations were observed (Table 3).

**Table 3:** Correlation analysis of sleep outcomes, race performance and cognitive performance N = 11.

	Δ2CRT Reaction Time*	Δ2CRT Throughput	Δ Interference Score	Sleep rating^a^*	Sleep rating^b^*	Total Distance
**Time in bed** ^a^	r = 0.38 [-0.31–0.80]	r = -0.51 [-0.85–0.13]	r = -0.18 [-0.70–0.48]	r = 0.07 [-0.57–0.65]	r = -0.01 [-0.62–0.61]	r = 0.07 [-0.55–0.64]
	p = 0.253	p = 0.112	p = 0.608	p = 0.847	p = 0.973	p = 0.837
**Time asleep** ^a^	r = -0.08 [-0.66–0.56]	r = 0.04 [-0.57–0.63]	r = -0.14 [-0.68–0.50]	r = 0.46 [-0.21–0.84]	**r = 0.62 [0.01–0.89]**	r = -0.46 [-0.83–0.20]
	p = 0.811	p = 0.905	p = 0.685	p = 0.156	**p = 0.041**	p = 0.157
**Sleep rating** ^a^ *	r = -0.40 [-0.81–0.28]	r = 0.31 [-0.37–0.78]	r = -0.01 [-0.62–0.61]	N/A	**r = 0.68 [0.11–0.91]**	r = -0.58 [-0.88–0.05]
	p = 0.222	p = 0.347	p = 0.989		**p = 0.022**	p = 0.061
**Time in bed** ^b^	r = 0.15 [-0.51–0.70]	r = -0.19 [-0.71–0.46]	r = -0.38 [-0.80–0.29]	r = 0.37 [-0.31–0.80]	r = 0.06 [-0.57–0.65]	r = 0.19 [-0.46–0.71]
	p = 0.670	p = 0.572	p = 0.249	p = 0.260	p = 0.852	p = 0.581
**Time asleep** ^b^ *	r = -0.21 [-0.73–0.46]	r = 0.09 [-0.54–0.66]	r = -0.18 [-0.70–0.47]	r = 0.43 [-0.25–0.82]	**r = 0.70 [0.16–0.92]**	r = -0.03 [-0.62–0.58]
	p = 0.537	p = 0.785	p = 0.602	p = 0.193	**p = 0.016**	p = 0.931
**Sleep rating** ^b^ *	**r = -0.64 [-0.9–0.4]**	**r = 0.61 [0.00–0.89]**	r = 0.24 [-0.44–0.74]	**r = 0.68 [0.11–0.91]**	N/A	r = -0.49 [-0.85–0.17]
	**p = 0.034**	**p = 0.045**	p = 0.473	**p = 0.022**		p = 0.124
**Total distance**	r = 0.25 [-0.43–0.75]	r = -0.10 [-0.66–0.53]	r = -0.26 [-0.74–0.41]	r = -0.58 [-0.88–0.05]	r = -0.49 [-0.85–0.17]	N/A
	p = 0.457	p = 0.769	p = 0.448	p = 0.061	p = 0.124	

Pearsons correlation.

*Spearman correlation.

^a^The night prior to the race.

^b^The 7-day average.

## 4. Discussion

This study examined the effects of running an ultra-marathon, including one night of sleep deprivation, on cognitive performance and its relationship with pre-race sleeping patterns. The principal findings from this study are: that (1) cognitive performance, in particular reaction time and executive function, was impaired after the race; and (2) there was a significant correlation between the perceived quality of sleep in the 7-days prior to the race and throughput in the 2CRT task. Collectively, these finding suggest that strategies to improve the quality of sleep in the week prior to an ultra-marathon could limit its negative impact on cognitive performance and improve race safety.

Sleep deprivation has been well documented to negatively impact cognitive performance, in both healthy volunteers and in athletes [5, 7]. High quality, empirical investigations on the cognitive effects of sleep deprivation on endurance sports are limited and contradictory. Solo offshore sailing [8], 5 hours of intermittent cycling [29] and a 168km non-stop ultra-marathon race [15] have each shown increases in reaction time post-event. Contrary to these results, Lucas and colleagues [27] found no significant change in reaction time following a 96–125 h continuous adventure race. However the sample size in the latter study was small (n = 9), perhaps reflecting the challenging nature of conducting any research at these types of events and this may help explain the heterogeneous findings [27]. Studies examining cognitive performance during ultra-marathons which do not include an element of sleep deprivation have not demonstrated any changes in reaction time [11, 12, 14]. Importantly, in this study, reaction time increased in the 2CRT (by 17%), the Stroop task (by 11%), and the Tower Puzzle (by 18%) after at least 16 hours of sleep deprivation.

Executive function, in particular response inhibition, has been found to be significantly impacted by sleep deprivation [30]. The Stroop task has been used extensively in the literature as a surrogate measure of executive function and the ability to exhibit behavioural self-control by assessing an individuals’ ability to ignore a learned response to enact a different action [11]. Existing literature on endurance events has reported no difference in the Stroop task following completion of a 96-125h adventure race [27], a 118km ultra-marathon [12] and 100km night run [11]. Contrary to these results we demonstrated a statistically significant 19% decrease in the Interference Score following completion of the race (Table 1). However, both ultra-marathons studied previously were shorter in distance (118 and 100 km respectively compared to a mean distance of 158.5 km in this study), did not report any sleep data, and were likely to involve less sleep deprivation, which may explain the divergent findings [11, 12]. Interestingly, decrements in the Digital Symbol Substitution Task and the 10-min serial response time tests, which both assess executive function, have also been reported after the North-Face UltraTrail du Mont-Blanc (UTMB^®^) 168 km race [15, 16]. The currents findings suggest that some aspects of cognitive performance, in particular reaction time and executive function, are negatively impacted by long endurance events which involve an element of sleep deprivation (>16 hours). It is also interesting to note that 62% of all adverse events occurred in the five participants with the greatest decline in cognitive performance, while only 15% of the adverse events occurred in the top five performing athletes (based on total distance covered). It is therefore plausible that runners could be at a greater risk of accidents due to an impaired ability to react quickly and to consider changes in their environment during similar events.

This study provides novel data to demonstrate a correlation between subjective pre-race sleep quality and cognitive performance during an ultra-marathon race. We demonstrated that higher quality sleep in the week prior to the race was associated with a smaller change in 2CRT reaction time and throughput following race completion compared to baseline measures. Athletes have been found to have poor sleep quality and quantity, while good sleep hygiene habits are associated with better sleep quality [35–38]. This, along with our current findings, suggests that improving sleep quality in the week prior to a race may mitigate the decrement in cognitive performance during an ultra-marathon. Unfortunately, there is currently limited data examining sleep strategies to minimise the cognitive impact of sleep deprivation in endurance events. Poussel and colleagues [18] demonstrated that pre-race sleep extension led to faster finish times in the UTMB 168 km ultra-marathon, while Hurdiel and colleagues [15] concluded sleeping for short periods during an ultra-marathon was an indication of severe fatigue and associated with poor cognitive performance as it did not allow for enough sleep to mitigate these effects. Several studies have assessed the effect of a sleep intervention on athletic performance in a range of sports [39], including tennis [40], basketball [36] and football players [41]. Sleep extension in the week prior to a sporting event was shown to improve physical performance and reaction time in basketball [36] and tennis players [40]. Roberts and colleagues [42] demonstrated sleep extension led to improved performance in endurance cycling by altering perceived effort, while Ritland and colleagues [43] showed sleep extension improved performance and executive function (using the Trail Making Test) in athletes undergoing military training. In our study most participants reported increasing their total time asleep in the week prior to the race with little variation in total sleep time between participants. While we found no statistical association between total time asleep and cognitive performance, perceived sleep quality was correlated with total sleep time. Sleep hygiene strategies in athletes have been shown to increase sleep duration, sleep quality and subjective sporting performance [41, 44, 45]. However, to date, no intervention studies have examined the impact of a pre-race sleep hygiene strategy on ultra-marathon running races which include an element of sleep deprivation. Further research is required to see if improving sleep quality prior to an ultra-marathon could lead to improved cognitive performance.

### 4.1 Limitations

This study was not without its limitations. Firstly, despite observing a significant effect on cognitive performance, with a large effect size, it is acknowledged that this is a small sample size. This also limits the feasibility of a multiple regression analysis assessing the impact of multiple variables in combination. Further research is required to confirm these findings in a larger sample. Secondly, despite attempting to recruit both males and females, only one female volunteered, and she did not reach the minimum time requirement to be included in the analyses. Thirdly, due to the nature of the race, there was a large variability in the distance covered by participants and in the amount of sleep deprivation experienced. However, all participants ran past 0300, which ensured they experienced an element of sleep deprivation. These limitations could be mitigated in future research by selecting an ultra-marathon with a set distance or completion time and recruiting more females. It is unclear how the current findings would translate to other endurance type events. While some studies have shown cognitive performance is dependent on testing time of day [16], there was no correlation in this study between any cognitive performance results and the time of day the tasks were undertaken. Finally, subjective sleep measures were used and no objective measures of sleep or physiological stress were measured. Future studies should consider the inclusion of non-invasive and objective measures of sleep (e.g. accelerometers).

## 5. Conclusions

This study indicates that cognitive performance is impaired when running an ultra-marathon which includes an element of sleep deprivation. In particular, reaction time and executive function deteriorated following race completion. The novel finding that good pre-race sleep quality was associated with a smaller decline in cognitive performance raises the possibility that improving sleep quality prior to a race could minimize these effects. Therefore, further research assessing the impact of a sleep quality intervention prior to a race could provide runners with an evidence-based strategy to improving race safety.

## Supporting information

S1 Checklist STROBE statement—Checklist of items that should be included in reports of observational studies. (DOCX)
